# Selective Gene Expression Analysis of Muscular and Vascular Components in Hearts Using Laser Microdissection Method

**DOI:** 10.1155/2012/863410

**Published:** 2012-06-12

**Authors:** Ayami Ikeda, Hisashi Kai, Hidemi Kajimoto, Suguru Yasuoka, Masayoshi Kage, Tsutomu Imaizumi

**Affiliations:** ^1^Division of Cardio-Vascular Medicine, Department of Internal Medicine, Kurume University School of Medicine, 67 Asahimachi, Kurume, Fukuoka 830-0011, Japan; ^2^Cardiovascular Research Institute, Kurume University, Kurume 830-0011, Japan; ^3^Department of Diagnostic Pathology, Kurume University Hospital, Kurume 830-0011, Japan

## Abstract

*Background*. The heart consists of various kinds of cell components. However, it has not been feasible to separately analyze the gene expression of individual components. The laser microdissection (LMD) method, a new technology to collect target cells from the microscopic regions, has been used for malignancies. We sought to establish a method to selectively collect the muscular and vascular regions from the heart sections and to compare the marker gene expressions with this method. *Methods and Results*. Frozen left ventricle sections were obtained from Wistar-Kyoto rats (WKY) and stroke-prone spontaneously hypertensive rats (SHR-SP) at 24 weeks of age. Using the LMD method, the muscular and vascular regions were selectively collected under microscopic guidance. Real-time RT-PCR analysis showed that brain-type natriuretic peptide (BNP), a marker of cardiac myocytes, was expressed in the muscular samples, but not in the vascular samples, whereas *α*-smooth muscle actin, a marker of smooth muscle cells, was detected only in the vascular samples. Moreover, SHR-SP had significantly greater BNP upregulation than WKY (*P* < 0.05) in the muscular samples. *Conclusions*. The LMD method enabled us to separately collect the muscular and vascular samples from myocardial sections and to selectively evaluate mRNA expressions of the individual tissue component.

## 1. Introduction

The heart consists of the cardiac muscle, vasculature, and to a lesser extent interstitial infiltrating cells. It is considered that the gene expressions are separately regulated in these tissue components under physiological and diseased conditions [1, 2]. Although the mRNA expression analysis has been established in the whole myocardium, it is difficult to investigate the expression level of each tissue component.

Recently, the laser microdissection (LMD) method has been developed to isolate specific microscopic regions from tissue samples and separately collect the specimens of interest, which enables us to selectively evaluate the mRNA expression levels in targeted cell clusters in the tissues, especially in malignant tissues [3]. The regions of interest are marked on the monitor of a vertical microscope and cut out by the laser beam under computer control. The isolated samples fall down into collecting tubes, which are subjected to quantitative real-time reverse-transcribed polymerase chain reaction (RT-PCR) or gene-chip/microarray assay. However, the LMD method has not been applied to the heart. Thus, we sought to establish a method to selectively collect the muscular region and arterial region in myocardial sections using the LMD method. The mRNA expression levels of maker genes specific for cardiac myocytes or vascular smooth muscle cells (VSMCs) were analyzed in the muscular and vascular samples, respectively, obtained from rat heart sections.

## 2. Methods

The study protocol was reviewed and approved by the Animal Care and Treatment Committee of Kurume University. Male Wistar-Kyoto rats (WKY) and stroke-prone spontaneously hypertensive rats (SHR-SP) were purchased from SLC (Shizuoka, Japan) and housed under standard conditions of humidity, room temperature, and a 12 : 12-hour dark-light cycles. They were provided with free access to tap water and chow. 

### 2.1. Animals

At 24 weeks, blood pressure was measured using a tail-cuff sphygmomanometer (MK-2000ST, Muromachi, Tokyo, Japan), as described previously [4]. Thereafter, rats were anesthetized with intraperitoneal ketamine (50 mg/kg) and xylazine (10 mg/kg). Percentage of left ventricular fractional shortening was measured using an echocardiography equipped with a 10 MHz transducer (Aloka, Tokyo, Japan) [5–8]. The next day, rats were euthanized with an overdose of pentobarbital (100 mg/kg, intraperitoneally). After the rats were perfused with ice-cold saline (4°C) at 100 mmHg, the heart was removed. The left ventricle was snap-frozen in isopentane/dry ice, embedded in OCT compound, and sectioned with cryostat. The cryosections (7 *μ*m in thickness) were mounted on ice-cold PEN-slides (Leica Microsystems, Wetzlar, Germany).

### 2.2. Laser Microdissection Method

**Figure 1:**
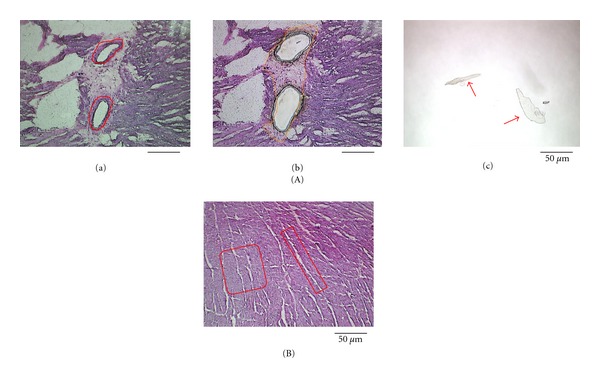
(A) Representative microphotographs demonstrating the sampling of vascular area of the myocardial section obtained from SHR-SP using LMD method. (a) Laser cut lines (red lines) were placed on the outside border of the medial smooth muscle layer. (b) Myocardial section after vascular samples were cut off by laser dissection shown. (c) After laser dissection, isolated vascular fragments (red arrows) fell down into the caps of Eppendorf tubes containing TRIzol reagent. The vascular fragments were being lysed in the TRIzol reagent. (B) For sampling myocardial area, laser cut lines were placed not to include microscopically visible vasculatures and infiltrating cells in the area of interest.

Fresh cryosections were fixed in RNase-free-ethyl acetate (acetic acid : ethanol = 1 : 19) and stained with 0.05% toluidine blue dissolved in RNase-free distilled water. The regions of the cardiac muscle and intramyocardial arteries were identified based on microscopic observation and were separately isolated from the section using LMD6000 system (Leica Microsystems). To collect vascular samples, we consistently placed the laser cut line on the outside border of the medial VSMC layer (Figure 1(A)). The isolated fragments were collected in the cap of an Eppendorf tube (Eppendorf Japan, Tokyo, Japan) containing TRIzol reagent (Life Technologies Japan, Tokyo, Japan). Muscular samples were dissected from the myocardium area without microscopically visible vasculatures and infiltrating cells (Figure 1(B)). The vascular sample included 40 cross-sections of the intramyocardial arteries for each animal. The muscular fragments with a total of 6 × 10^4^ *μ*m^2^ were collected in each animal.

### 2.3. Quantitative Real-Time RT-PCR

**Figure 2:**
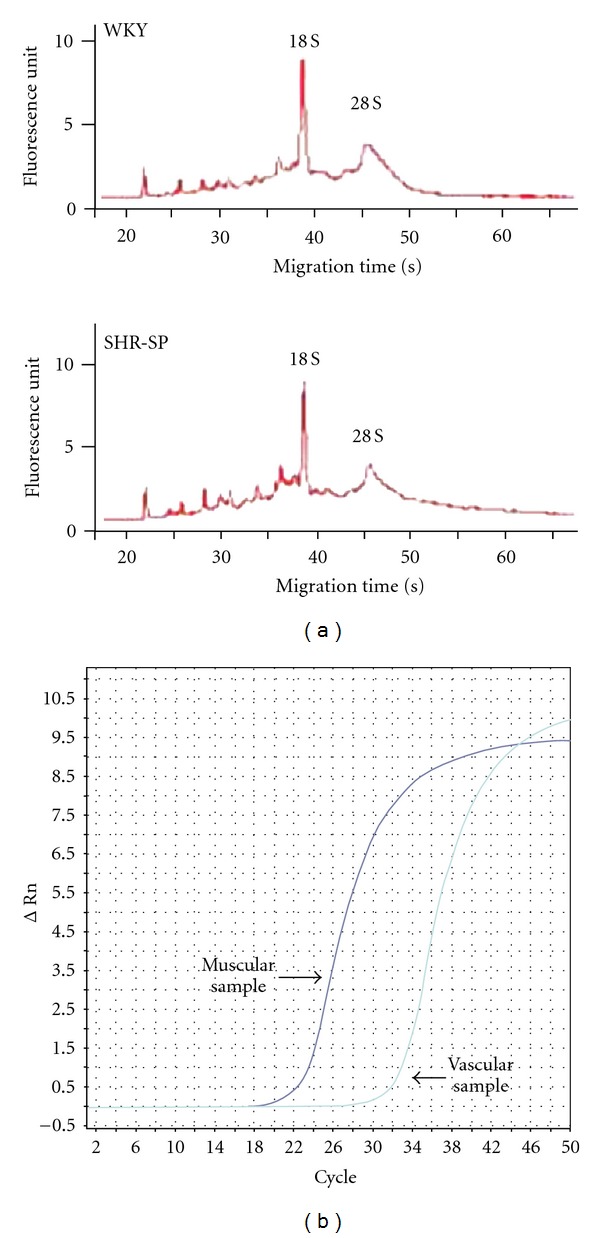
(a) Representative RNA electrophoregrams of the muscular samples of WKY (top) and SHR-SP (bottom). (b) Representative amplification plots of BNP mRNA in the muscular and vascular samples of WKY.

Total RNA was purified using RNeasy micro (Qiagen, Valencia, CA) according to the manufacturer's instructions. Electropherogram exhibited clear peaks for 18S and 28S ribosomal RNAs in the purified RNA samples (Figure 2(a)). RNA was reverse transcribed using a High Capacity RNA-to-cDNA kit (GE Health Care, Waukesha, WI). Equal amount of the resulting cDNA was subjected to real-time PCR using the TaqMan Universal PCR Master Mix and a Sequence Detection System model 7700 (Life Technologies Japan) [5, 9, 10]. Primer pairs and TaqMan probes for rat type B-natriuretic peptide (BNP), *α*-smooth muscle actin (*α*-SMA), and *β*-actin were obtained from GE Health Care. Good amplification plots for target genes were shown in the muscular and vascular samples (Figure 2(b)). Expression level of the target gene was normalized by *β*-actin level in each sample.

### 2.4. Statistical Analysis

Data were expressed as mean ± SD. Unpaired *t*-test was performed for comparison between two groups.

## 3. Results

**Figure 3:**
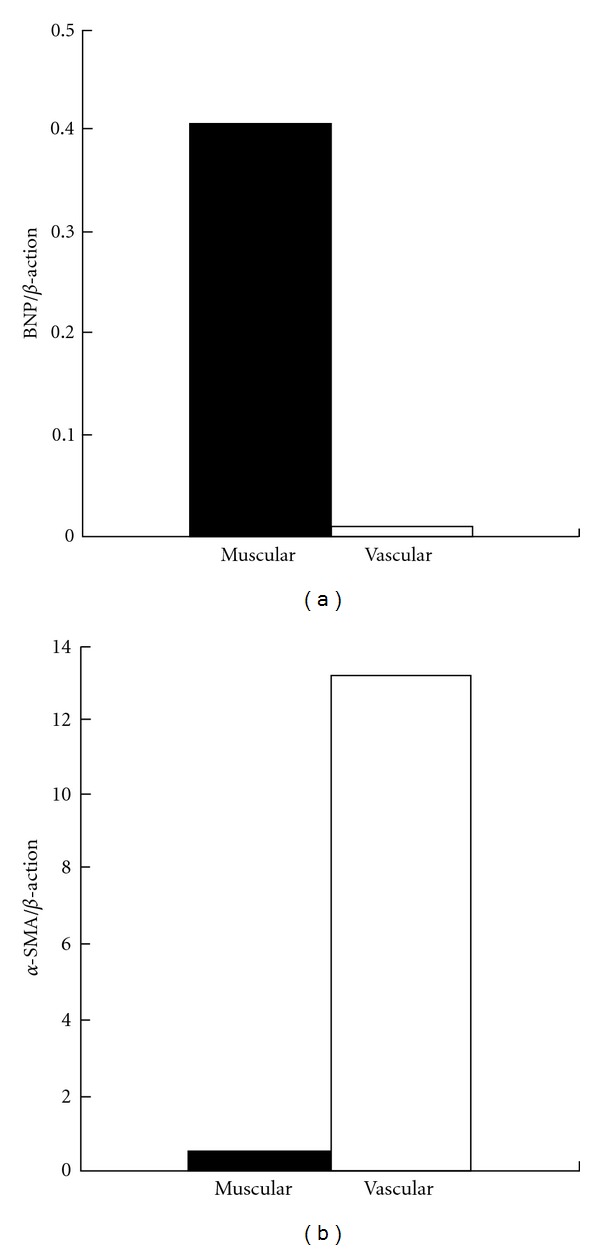
BNP and *α*-SMA mRNA expression in the muscular (closed column) and vascular (open column) samples obtained from the heart of WKY (*n* = 2). Expression level of the target gene was normalized by *β*-actin level in each sample.

**Figure 4:**
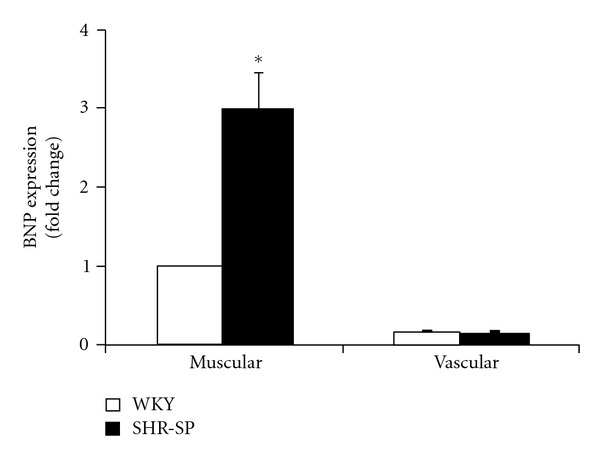
BNP mRNA expression in the muscular and vascular samples obtained from the heart of WKY (open column) and SHR-SP (closed column). BNP expression levels were expressed as fold change from the muscular sample of WKY. Unpaired *t*-test was used for comparison of initial data before expression as fold changes. Bar = 1 × SD (*n* = 3). *P < 0.05 versus WKY.

At 24 weeks of age, systolic blood pressure was 120.5 ± 12.2 mmHg in WKY (*n* = 5) and 252.5 ± 16.7 mmHg in SHR-SP (*n* = 3) (*P* < 0.001). Percentage of Left ventricular fractional shortening was 26.8 ± 4.1% in WKY (*n* = 5) and 29.8 ± 5.2% in SHR-SP (*n* = 3) (no significance). We present one example of real-time RT-PCR analysis in WKY, which showed BNP mRNA expression in the muscular samples, but not in the vascular samples (Figure 3). In contrast, *α*-SMA expression was found exclusively in the vascular samples. Next, we compared the expression levels of BNP, a molecular marker of cardiac myocyte hypertrophy, between WKY and SHR-SP (Figure 4). SHR-SP had a significantly greater BNP expression than WKY in the muscular samples. BNP mRNA was not expressed in the vascular samples of WKY and SHR-SP. 

## 4. Discussion

The present study demonstrated that the LMD method enabled us to selectively collect myocardium and intramyocardial arteries from the heart section. Selective sampling was verified because the expression of cardiac myocyte-specific gene marker, BNP, was detected exclusively in the muscular samples, whereas the VSMC-specific marker, *α*-SMA, was expressed only in the vascular samples. Moreover, hypertrophic gene upregulation, as assessed by BNP expression, was significantly greater in the muscular samples obtained from SHR-SP than from WKY.

Recently, it has been reported that the LMD method is useful for selective sampling of the arterial component from the surrounding tissues, for example, the isolation of the arterial lesions in the lung specimen of patients with familiar pulmonary hypertension [11] and the isolation of the collateral vessels in the ischemic hindlimb in mice [12]. Also, the LMD method was used for selective sampling of the intimal plaques in the human atherosclerotic lesions [13, 14]. However, there have been few studies using the LMD method for gene expression analysis of the heart [15]. Thus, we sought to establish the method to selectively collect muscular and vascular samples from the heart sections using the LMD method.

As shown in Figure 1(A), the intramyocardial arterioles were surrounded by thin loose connective tissue separating from the cardiac muscle tissue. Thus, the microscopic guidance allows us to easily isolate vascular samples. In this study, we successfully collected and analyzed the intramyocardial arteries with a diameter of approximately 50 *μ*m. This finding was in line with the previous studies demonstrating that relatively small vascular samples, such as intrapulmonary arteries at a size between 50 and 200 *μ*m and intrarenal arterioles with a diameter of approximately 100 *μ*m, were selectively isolated using the LMD method [16, 17]. In contrast, it was necessary that we consistently took a great care in isolating muscular samples by avoiding microscopically visible vessels and infiltrating cells. The mRNA expressions of BNP and *α*-SMA were not detected in the vascular and muscular samples, respectively (Figure 3). Moreover, the BNP mRNA upregulation associated with cardiac hypertrophy was documented specifically in the muscular samples in SHR-SP (Figure 4). These findings suggested that the contamination of cardiac myocytes in the vascular samples or VSMCs in the muscular samples was negligible.

In conclusion, the LMD method enabled us to separately collect the muscular and vascular samples from the myocardial sections and to selectively evaluate the mRNA expression changes in individual tissue component.
